# Comparison of pathological features of patients with antibody-negative and positive primary biliary cholangitis

**DOI:** 10.1186/s12876-025-04280-5

**Published:** 2025-09-29

**Authors:** Hong-Li Liu, Xing Liu, Yi-Fan Hu, Xi-Xuan Wang, Yu Zhang, Li Wang, Sha-Sha Li, Yi-Jun Bao, Si-Wei Zheng, Qing-Fang Xiong, Yan-Dan Zhong, Du-Xian Liu, Ping Huang, Xiao-Ning Feng, Wen-Quan Zeng, Cai-Yun Zhang, Kai Zhang, Yong-Feng Yang

**Affiliations:** 1 Department of Infectious Disease and Liver Disease, The Second Hospital of Nanjing, Teaching Hospital of Southeast University, Nanjing, 210003 China; 2 Clinical Research Center, The Second Hospital of Nanjing, Affiliated to Nanjing University of Chinese Medicine, Nanjing, 210003 China; 3 Department of Infectious Disease and Liver Disease, The Second Hospital of Nanjing, Affiliated to Nanjing University of Chinese Medicine, Nanjing, 210003 China; 4 Department of Hepatology, The Second Hospital of Nanjing, Clinical Teaching Hospital of Medical School, Nanjing University, Nanjing, 210003 China; 5 Department of Pathology, The Second Hospital of Nanjing, Affiliated to Nanjing University of Chinese Medicine, Nanjing, 210003 China

**Keywords:** Primary biliary cholangitis, Pathological features

## Abstract

**Objective:**

To examine the differences and similarities among primary biliary cholangitis (PBC) with positive and negative expression of autoantibodies (anti-mitochondrial antibodies AMA and AMA-M2, antinuclear antibodies anti-gp210, and anti-sp100).

**Methods:**

Clinical and pathological data of 85 PBC in patients who underwent liver puncture biopsy were retrospectively collected from January 2016 to September 2018 at the Second Hospital of Nanjing. The differences in clinical and pathological indexes of PBC between autoantibody negative and positive groups were analyzed by SPSS 25.0, and the patients’ previous autoantibody indexes were collected for follow-up.

**Results:**

The average age of 85 PBC patients was (52.2 ± 9.2) years old, including 15 males (17.6%) and 70 females (82.4%), with a male-to-female ratio of 1:4.7. Fifty-nine autoantibody-positive cases and 26 autoantibody-negative cases were 50 (84.7%) AMA positive, 51 (86.4%) AMA-M2 positive, 21 (35.6%) anti-gp210 positive, and 15 (25.4%) anti-sp100 positive. The distribution of CK7-positive hepatocytes and the degree of bile duct injury in the portal area of PBC patients in the antibody-negative group were lower than those in the antibody-positive group (*P* < 0.05). There was no significant difference in the distribution of interface inflammation, plasma cell grade, ductular reaction, and fibrosis between the two groups. When the autoimmune antibodies were reexamined at 2 months–7 years (median time 1 year), AMA, AMA-M2, anti-gp210, and anti-sp100 were still negative in 16 antibody-negative patients.

**Conclusions:**

The pathological symptoms and diagnostic patterns of autoantibody-negative and positive PBC were generally comparable. In this study, the pathological manifestations of antibody-negative PBC were similar to those of antibody-positive PBC, but the lymphocyte aggregation in antibody-negative PBC was more severe than that in antibody-positive PBC, while the degree of bile duct injury and CK7-positive hepatocytes were milder than that in antibody-positive PBC, suggesting that the overall pathological changes of antibody-negative PBC were slightly milder than those of antibody-positive PBC.

## Introduction

Primary biliary cholangitis (PBC) is chronic intrahepatic cholestasis characterized by persistent, nonsuppurative, destructive small bile duct inflammation that may lead to cirrhosis eventually [1, 2]. It frequently affects small bile ducts less than 100 μm in diameter. Anti-mitochondrial antibody (AMA) is an important serological marker for diagnosing PBC. The target antigens are distributed in mitochondria, and they are divided into 9 types (M1-M9) according to the location of AMA target antigen in the membrane of mitochondria, its sensitivity to trypsin, and electrophoretic characteristics. M2, M4, and M9 subtypes relate to PBC. Now, antinuclear antibodies anti-gp210 and anti-sp100 are also serologic indicators for diagnosing PBC [1], and pathological liver examination is necessary to confirm the diagnosis of autoantibody-negative PBC [3]. However, patients with typical pathological diagnosis patterns are more consistent with the characteristics of PBC. Alagille syndrome, progressive familial intrahepatic cholestasis, and other cholestatic diseases should be excluded from diagnosing PBC. Studies have debated whether there are clinicopathological differences between AMA-positive and AMA-negative PBC.

In this study, the pathological manifestation pattern was added to assess the pathological features of PBC, which are dominated by bile duct damage and granuloma or lymphocyte aggregation of antibody-negative individuals, to compare the pathological characteristics of antibody-positive and negative PBC.

## Method

### Research subject

Retrospective data was gathered on PBC outpatients or inpatients who had liver puncture biopsies at the Second Hospital of Nanjing between January 2016 and September 2018. Eighty-Five PBC patients were included, the Medical Ethics Committee of the Second Hospital of Nanjing approved the study (No. 2020-LS-ky030). In this retrospective study, the data were anonymized, and the requirement for informed consent was waived by the Medical Ethics Committee of the Second Hospital of Nanjing. This study was approved by the examination of the Second Hospital of Nanjing. The study protocol conformed to the ethical guidelines of the Declaration of Helsinki.

### Inclusion and exclusion criteria

#### Inclusion criteria

Patients satisfied the diagnostic criteria for PBC in the 2021 recommendations for diagnosing and treating primary biliary cholangitis [1].

#### Exclusion criteria

(1) Portal areas of liver puncture pathological tissue were less than five; (2) The data of anti-AMA, AMA-M2, anti-gp100 and anti-sp100 could not be traced back; (3) Combined with active viral hepatitis; (4) For antibody-negative PBC patients, pathological diagnosis combined with follow-up evaluation was applied to carefully exclude other disorders such as viral hepatitis, Progressive Familial Intrahepatic Cholestasis type 3, and Alagille syndrome, IgG4-hepatopathy.

### Data collection and pathological section reading

Clinical data were collected, such as gender, age, clinical manifestations, liver function, immunoglobulin, AMA, AMA-M2, anti-sp100, and anti-gp210. AMA was detected by indirect immunofluorescence assay (IFA) using the EUROIMMUN Autoantibody Profile IgG kit, with rat kidney tissue as the substrate. A dilution of 1:100 was defined as the cut-off for positivity. AMA-M2, anti-gp210, and anti-sp100 were detected using the EUROIMMUN EUROLINE immunoblot assay (EUROIMMUN, Germany), and a visible band was regarded as positive. All results were independently reviewed by two laboratory physicians.

Pathological data of hematoxylin–eosin (HE) and Masson staining, the immunohistochemistry staining of CD38 and CK7. The liver specimens were analyzed by two clinicians. They had the pathological experiences and can evaluate the patient’s pathological characteristic without the information of patients.

1. In each pathological slides, the number of complete or incomplete portal areas (an incomplete portal area was defined as a boundary with more than 2/3 of the portal area). And the number of portal areas should analyze the following pathological features: lymphocytic aggregates of portal areas (present or absent), epithelioid granuloma (present or absent), and bile duct injury (none, mild, moderate, absent) Table 1.
2. Staging

**Table 1:** Pathological scores of PBC patients

Features	Score
Fibrosis	
Absence of fibrosis	0
Fibrosis was confined to the portal areas	1
Fibers extended from the portal area but did not form a bridge	2
Bridging fibrosis was seen, but lobular structures were still present	3
Pseudolobules were formed	4
Interface inflammation	
None: boundary plate intact	0
Mild: boundary plate destruction < 30%	1
Moderate: 30% to 50% destruction of the boundary plate	2
Severe: 50% destruction of the boundary plate	3
The proportion of bile duct missing	
< 1/4	1
1/4-1/2	2
1/2-3/4	3
> 3/4	4
Plasma cells in CD38	
None: no plasma cells	0
Few: scattered positive cells	1
Middle: positive in the cluster	2
Moderate: diffusely positive	3
Ductular reaction	
None: No ductular reaction was observed	0
Less: ductular reaction in portal area < 1/2	1
Mostly: ductular reaction in portal area ≥ 1/2	2
CK7 positive in hepatocytes	
None: No CK7 was positive in hepatocytes around the portal area	0
Less: 1/3 positive hepatocytes around the portal area;	1
Medium: between 1 and 3;	2
More: more than 2/3 of the portal area surrounding hepatocytes were positive	3
Degree of bile duct injury	
None: No bile duct injury was observed	0
Mild: bile duct cells arranged slightly disordered, accompanied by lymphocyte infiltration	1
Obvious: The arrangement of bile duct cells or the luminal structure was disordered, but the bile duct was visible	2
Absence: No accompanying bile ducts were seen beside the arterioles in the portal area	3

The histopathological features of liver puncture pathology were scaled by the semi-quantitative scoring system, including fibrosis, interfaces, the percentage of absent bile ducts, plasma cells, bile ductular reaction, and CK7-positive hepatocytes (Table 1). Pathological staging was based on Nakanuma’s [2] modified PBC staging criteria (Table 2), the grade of CK7-positive hepatocytes grade was substituted for evaluating copper-positive in Orcein staining to assess the level of cholestasis.

**Table 2:** Based on the Japanese Nakanuma PBC score combined with CK7 hepatocytes and staging

Score	pathological feature
Fibrosis	
0	There was no fibrosis or fibrosis was confined to the portal area
1	Fibrosis in the portal area extended into the lobules, occasionally forming incomplete septa
2	Fully connected fibrous septa were accompanied by disorganization of the lobular architecture
3	Cirrhosis (extensive fibrosis with pseudolobular formation)
Bile duct defect	
0	The intralobular bile ducts in the portal area could be distinguished
1	Absence of bile duct was found in 1/3 portal area
2	One third to two thirds of the portal area showed absence of bile duct
3	More than two thirds of the portal area showed absence of bile duct
Cholestasis (CK7 positive in hepatocytes)	
0	There was no CK7 expression in the hepatocytes around the portal area
1	Hepatocytes were positive around 1/3 portal area
2	Between 1 and 3
3	More than 2/3 of the portal areas were positive for hepatocytes
Grading	
0	Stage 1
1–3	Stage 2 (mild progression)
4–6	Stage 3 (moderate progression)
7–9	Stage 4 (severe progression)

### Follow-up

Autoantibody alterations in the negative group were collected before and after hospitalization for analysis and follow-up ended in August 2022.

### Data analysis

The data were processed and analyzed with SPSS 25.0 and GraphPad 8.0. The values of measurement data that were normally distributed were described by (mean ± standard deviation) and analyzed by t-test; measurement data that were not normally distributed were analyzed by median (P_50_) and quartiles (P_25_, P_75_). Count data and rank data were described by cases and percentages, and Mann–Whitney U test, chi-square test, or Fisher exact test were conducted. A difference with a *P* < 0.05 was considered statistically significant.

## Results

### General information

In this study mean age of (52.2 ± 9.2) years, male-to-female of 1:4.7. Fifteen patients (17.6%) were men, and 70 patients (82.4%) were women. There were 59 patients in the autoantibody-positive group and 26 patients in the autoantibody-negative group. The percentage of antibody positives was 84.7% (50) for AMA, 86.4% (51) for AMA-M2, 35.6% (21) for anti-gp210, and 25.4% (15) for anti-sp100. The average age of the antibody-positive group was (52.0 ± 8.3) years, with 11 men (18.6%) and 48 women (81.4%), a male-to-female ratio of 1:4.4, and 30 people (50.8%) having overlapping autoimmune hepatitis (AIH); The average age of the antibody-negative group was (52.7 ± 11.2) years, with a male-to-female ratio of 1:5.5, 4 males (15.4%), and 22 females (84.6%). There were 13 overlapping AIH (50.0%); there were no statistically significant variations in age, gender, or the ratio of overlapping AIH (*P* > 0.05) Table 3.

**Table 3:** The Comparison between clinical manifestation and laboratory tests between antibody-negative and antibody-positive groups

Group Indexes	Antibody-negative N(%)/Median(P_25_,P_75_)	Antibody-positive N(%)/Median(P_25_,P_75_)	*P*
Overlap AIH	13(50.0%)	30(50.8%)	0.943
Fatigue	9(34.6%)	30(50.8%)	0.166
Pruritus	2(7.7)	1(1.7)	0.220
Jaundice	7(26.9)	22(37.3)	0.353
Edema	0(0)	1(1.7)	1.000
Liver function	(*n* = 26)	(*n* = 56)	
TB(μmol/L)	15.2(10.9,24.9)	16.2 (12.1,28.1)	0.684
DB(μmol/L)	6.0 (3.7,10.8)	5.9 (3.5,14.2)	0.174
ALT(IU/L)	69.4 (43.1,163.5)	57.4 (37.4,92.0)	0.790
AST(IU/L)	72.7 (40.3,149.5)	64.9 (41.8,105.9)	0.742
ALB(g/L)	40.0 (35.2,42.9)	40.5 (36.8,44.5)	0.705
GGT(U/L)	233.1(81.1,397.1)	193.4 (99.5,343.1)	0.889
ALP(IU/L)	185.7(135.5,403.8)	204.2 (129.1,294.6)	0.618
TA(μmo/L)	22.7 (8.1,53.6)	24.2 (12.4,46.0)	0.731
Immunglobulin	(*n* = 25)	(*n* = 59)	
IgA(g/L)	2.5 (2.0,3.0)	2.9 (2.1,3.9)	0.172
IgG(g/L)	14.4 (12.4,19.1)	15.3 (12.0,19.4)	0.678
IgM(g/L)	1.5 (1.0,2.5)	2.8 (2.2,4.4)	< 0.001^***^

*AIH* Autoimmune Hepatitis, *TB* total bilirubin, *DB* direct bilirubin, *ALT* alanine aminotransferase, *AST* aspartate aminotransferase, *ALB* albumin, *GGT* gamma-glutamyl transferase, *ALP* alkaline phosphatase, *TA* total bile acids, *IgA* immunoglobulin A, *IgG* immunoglobulin G, *IgM* immunoglobulin M

^***^, *P* < 0.001

### Clinical manifestations and laboratory tests

Fatigue was the most common symptom in both groups. The main clinical features of the antibody-negative and antibody-positive groups are summarized in Table 3. No significant differences were observed between the two groups (all *P* > 0.05).

There were no statistically significant differences in IgA, IgG, or liver function (*P* > 0.05), but the IgM level in the antibody-negative group was lower than that in the positive PBC group (*P* < 0.001) (Table 3).

### Pathological traits

#### Pathological features of HE staining

There was no statistical difference between the antibody-negative and antibody-positive groups in terms of pathological aspects such as degree of interface inflammation, fibrosis, ductular reaction, ductopenia, and CD38 plasma cell infiltration; however, the grade of CK7-positive hepatocytes in the antibody-negative group (0–3) was lower than the grade distribution in the antibody-positive group, and the difference was statistically different (*P* < 0.05) (Table 4).

**Table 4:** Comparison of the number of cases with pathological characteristics of PBC patients between antibody negative and positive groups

Pathological features and grades		Antibody negative (*n* = 26) N,(%)	Antibody positive(*n* = 59) N,(%)	*P*
Interface Inflammation	0	3(11.5)	9(15.3)	0.741
	1	10(38.5)	20(33.9)	
	2	11(42.3)	19(32.2)	
	3	2(7.7)	11(18.6)	
Fibrosis	0	0(0)	0(0)	0.386
	1	4(15.4)	20(33.9)	
	2	12(46.1)	17(28.8)	
	3	8(30.8)	16(27.1)	
	4	2(7.7)	6(10.2)	
Ductular reaction	0	10(38.4)	16(27.1)	0.721
	1	8(30.8)	28(47.5)	
	2	8(30.8)	15(25.4)	
CK7-positive hepatocytes	0	17(65.4)	24(40.7)	0.015^*^
	1	8(30.8)	21(35.6)	
	2	1(3.8)	14(23.7)	
	3	0(0)	0(0)	
Ductopenia	1	13(50.0)	22(37.3)	0.368
	2	5(19.2)	18(30.5)	
	3	8(30.8)	15(25.4)	
	4	0(0)	4(6.8)	
Plasma cells	0	2(7.7)	2(3.4)	0.842
	1	12(46.1)	31(52.5)	
	2	8(30.8)	21(35.6)	
	3	4(15.4)	5(8.5)	

(^*^ P < 0.05)

#### Pathological staging characteristics

The antibody-negative group made up 61.5% of stage 1–2 and 38.5% of stage 3–4, according to the modified pathological staging combined with the Japanese researcher Nakanuma, while the antibody-positive group made up 49.2% of stage 1–2 and 50.8% of stage 3–4. Despite the milder clinical phases in the antibody-negative group, there was no statistically significant difference (*P* = 0.181) (Table 5).

**Table 5:** Comparison of pathological stages of PBC patients with Nakanuma-modified CK7-positive hepatocytes between antibody-negative and positive groups

	1 N,(%)	2 N,(%)	3 N(%)	4 N(%)	Total N	*P*
Antibody-negative	3(11.5)	13(50.0)	9(34.6)	1(3.9)	26	0.181
Antibody-positive	2(3.4)	27(45.8)	26(44.0)	4(6.8)	59	

#### Pathological characteristics portal area count

**Fig. 1:**
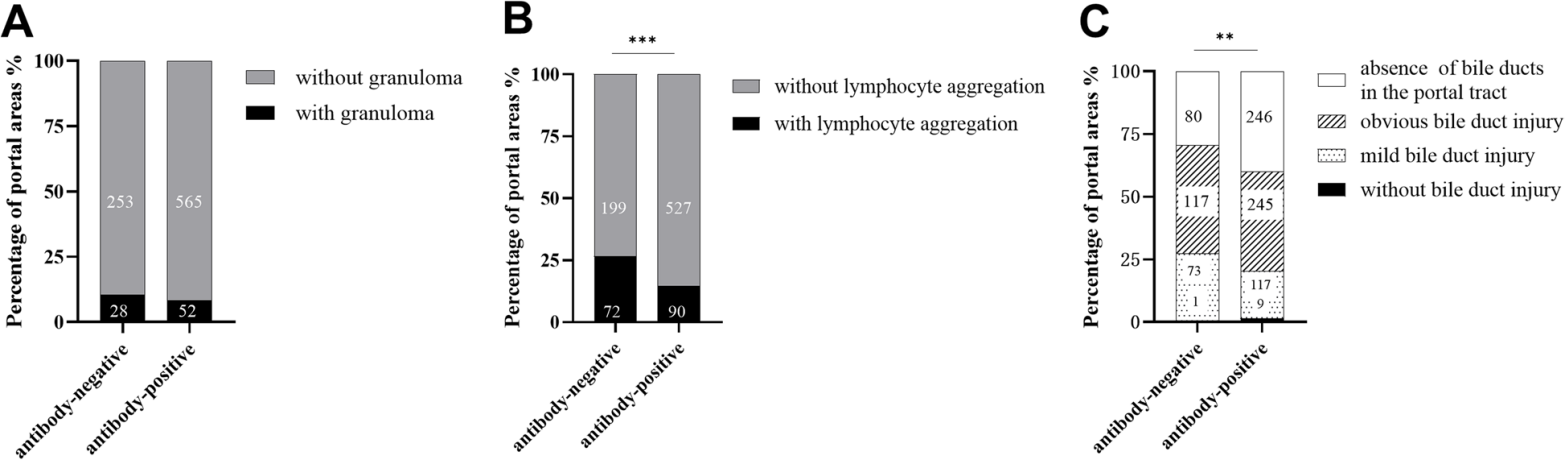
**A**-**B**-**C** Comparison of the pathological features of PBC in counting granuloma of the portal area, lymphocyte aggregation, and bile duct injury between the 26 antibody-negative groups and 59 positive groups (*** *P* < 0.001,** *P* < 0.01)

In this study, there were a total of 271 and 617 portal areas in 26 antibody-negative and 59 antibody-positive patients, respectively. There was no statistically significant difference in the amount of epithelioid granuloma in the portal areas (*P* > 0.05), but there was (*P* < 0.05) in the degree of lymphocytic aggregation and bile duct injury (Fig. 1A-B-C). Bile duct injury was found in both groups. 92.3% of the patients in the antibody-negative group had lymphocyte aggregation and/or epithelioid granuloma based on bile duct injury, and 89.4% of the patients in the antibody-positive group had lymphocyte aggregation and/or epithelioid granuloma based on bile duct injury (*P* > 0.05) (Table 6).

**Table 6:** Comparison of the number of cases with pathological patterns between antibody-negative and antibody-positive patients

Pathologic feature	Number of antibody-negative cases (*n* = 26) Number of cases and constituent ratio (%)	Number of antibody-positive cases (*n* = 59) Number of cases and constituent ratio (%)	Total	*P*
Bile duct injury only	2(7.7)	7(11.9)	9(10.6)	0.240
Lymphocyte aggregation + bile duct injury	5(19.2)	22(37.3)	27(31.8)	
Granuloma + bile duct injury	1(3.8)	1(1.7)	2(2.4)	
Lymphocyte aggregation + granuloma + bile duct injury	18(69.3)	29(49.1)	36(55.2)	

### Follow-up in antibody-negative PBC

Sixteen of the 26 patients with PBC in the antibody-negative group were retested for autoimmune antibodies AMA, AMA-M2 antibody, anti-gp210, and anti-sp100 before and after hospitalization, with a time interval between retesting ranging from 2 months to 7 years (median was 1 year), and none of the 16 patients experienced any change of the above antibodies to positive.

## Discussion

PBC is a chronic intrahepatic cholestatic disease typically manifests pathologically as chronic non-suppurative destructive cholangitis. As the disease progresses, it can also cause liver fibrosis and cirrhosis. Similar to other studies [3–6], the similarities and differences between antibody-negative and positive PBC were observed in this study. Some studies believe the AMA negative and positive should be no significant differences in the clinical, morphological, and laboratory data of AMA-positive and AMA-negative forms of PBC in the mechanisms [7, 8]. However, the proportion of antibody-negative patients in our study (26/85, 30.5%) is indeed higher than that reported in previous studies (5%−10%). Because we have differences in patient enrollment, this was a retrospective study, and all cases were confirmed by liver biopsy and further validated through follow-up, which confirmed the diagnosis of PBC. In clinical practice, antibody-positive patients are usually diagnosed without biopsy, and those undergoing biopsy are typically patients with diagnostic difficulties or poor treatment response; therefore, they were less frequently included in our biopsy-based cohort. In contrast, antibody-negative patients must undergo liver biopsy for confirmation, which led to a relatively higher proportion of antibody-negative PBC in our study. This explains the lower AMA positivity rate. The pathological manifestation pattern was added in this study to assess the pathological features of PBC, which are dominated by bile duct damage and granuloma or lymphocyte aggregation, and the diagnosis of PBC in antibody-negative individuals. Alagille syndrome, progressive familial intrahepatic cholestasis-3, and other cholestatic disease etiologies should be ruled out before diagnosing PBC in antibody-negative patients with just pathological bile duct damage.

In the early stage of PBC, the bile duct is slightly damaged, the shape and size of biliary epithelial cells are irregular, or the nuclei are condensed. The bile duct structure progressively degrades and vanishes as more lymphocytes surround the residual bile ducts, which release phospholipid-like substances and draw nearby phagocytes to form epithelioid granulomas. The distribution of CK7 positive hepatocytes was fewer in the autoantibody-negative group than in the positive group, indicating that cholestasis was severe in the antibody-positive group. CK7^+^ hepatocytes were related to cholestasis. CK7^+^ hepatocytes occur in the canals of Hering, the site of transition of hepatocytes into biliary epithelial cells, where hepatocytes transition into biliary epithelial cells. During proliferation, the degree of CK7-related cell differentiation may differ between antibody-positive and antibody -negative PBC. The bile duct injury in the positive group was more severe than in the negative group, and the serum IgM-positive group was higher, suggesting that the degree of pathological injury in antibody-negative PBC was slightly less than that in antibody-positive PBC. More lymphocytes were accumulated in the negative group, which may indicate that more lymphocytes were recruited by exudating the damaged bile duct in the early stage of bile duct injury. As for the clinic, the CK7 and CD38 is routine in this pathology test, the two indexes can show the bile duct damage and plasma cells, which can help us distinguish the features of PBC.

The proportion of stage 3–4 PBC patients in the antibody-positive group (50.8%, 30/59) was higher than in the antibody-negative group (38.5%, 10/26) in terms of pathological staging, but there was no statistically significant difference between the two groups. This may have been due to the small number of antibody-negative PBC cases included in this study. The commonly used pathological staging of PBC is Scheuer [9] and Ludwig [10] staging, which only reflects the severity of inflammation and fibrosis in the portal area and does not include the degree of bile duct injury, and is highly subjective. For fibrosis, bile duct loss, and cholestasis, Nakanuma’s stage ratings are more objective and accurately reflect the PBC’s overall pathological alterations. The pathological staging based on this scholar’s staging was used in this study to evaluate the characteristics of PBC because cholestasis evaluation requires an orcein granule staining method on frozen specimens, and some studies suggest that CK7-positive hepatocytes are positively correlated with copper staining [11].

However, the discussion in this paper, including the impossibility of comparing pathological features in positive and negative autoantibody PBC groups with the same clinical stage owing to the small numbers. But if we can distinguish the stage. the proportion of stage 3–4 PBC patients in the antibody-positive group (50.8%, 30/59) was higher than in the antibody-negative group (38.5%, 10/26) in terms of pathological staging, but there was no statistically significant difference between the two groups. If we can increase the number, we may Foung the positive has the significance.

## Conclusion

In conclusion, the pathological manifestations of antibody-negative PBC and antibody-positive PBC in this study were similar, but the lymphocyte aggregation was more severe, while the bile duct injury and CK7 positive hepatocytes were less severe in antibody-negative PBC than in antibody-positive PBC, suggesting that the overall pathological changes of antibody-negative PBC were slightly milder than those of antibody-positive PBC. During the follow-up, AMA, AMA-M2, anti-gp210 and anti-sp100 antibodies did not turn positive in antibody-negative PBC patients, suggesting that antibody-negative PBC and antibody-positive PBC may have different immune mechanisms. This study is a retrospective study with a small sample size, and prospective, multi-center studies are needed to further confirm the pathological differences between antibody-negative and positive PBC.

## Data Availability

The datasets used and analyzed during the current study are available from the corresponding author on request.
